# Inhibition of a Descending Prefrontal Circuit Prevents Ketamine-Induced Stress Resilience in Females

**DOI:** 10.1523/ENEURO.0025-18.2018

**Published:** 2018-03-06

**Authors:** S. D. Dolzani, M. V. Baratta, J. M. Moss, N. L. Leslie, S. G. Tilden, A. T. Sørensen, L. R. Watkins, Y. Lin, S. F. Maier

**Affiliations:** 1 Department of Psychology and Neuroscience and the Center for Neuroscience, University of Colorado Boulder, Boulder, CO 80309; 2 Institute for Behavioral Genetics, University of Colorado Boulder, Boulder, CO 80309; 3 Department of Neuroscience, University of Copenhagen, Copenhagen, 1165 Denmark; 4 McGovern Institute for Brain Research, Department of Brain and Cognitive Sciences, Massachusetts Institute of Technology, Cambridge, MA 02139

**Keywords:** dorsal raphe nucleus, ketamine, prefrontal cortex, resilience, serotonin, stress

## Abstract

Stress is a potent etiological factor in the onset of major depressive disorder and posttraumatic stress disorder (PTSD). Therefore, significant efforts have been made to identify factors that produce resilience to the outcomes of a later stressor, in hopes of preventing untoward clinical outcomes. The NMDA receptor antagonist ketamine has recently emerged as a prophylactic capable of preventing neurochemical and behavioral outcomes of a future stressor. Despite promising results of preclinical studies performed in male rats, the effects of proactive ketamine in female rats remains unknown. This is alarming given that stress-related disorders affect females at nearly twice the rate of males. Here we explore the prophylactic effects of ketamine on stress-induced anxiety-like behavior and the neural circuit-level processes that mediate these effects in female rats. Ketamine given one week prior to an uncontrollable stressor (inescapable tailshock; IS) reduced typical stress-induced activation of the serotonergic (5-HT) dorsal raphe nucleus (DRN) and eliminated DRN-dependent juvenile social exploration (JSE) deficits 24 h after the stressor. Proactive ketamine altered prelimbic cortex (PL) neural ensembles so that a later experience with IS now activated these cells, which it ordinarily would not. Ketamine acutely activated a PL to DRN (PL-DRN) circuit and inhibition of this circuit with Designer Receptors Exclusively Activated by Designer Drugs (DREADDs) at the time of IS one week later prevented stress prophylaxis, suggesting that persistent changes in PL-DRN circuit activity are responsible, at least in part, for mediating long-term effects associated with ketamine.

## Significance Statement

Stress-related disorders affect females at twice the rate of males, and so identifying factors capable of producing resilience in females is critically important. Recent efforts to identify neural mechanisms underlying the prophylactic effects of ketamine on the behavioral and neural impact of a later stressor have focused solely on male rodents. Here we show that ketamine administered to female rats one week before an uncontrollable stressor prevents stress-induced anxiety-like behavioral effects. The mechanisms by which ketamine exerts prophylactic effects were explored, and ketamine was found to activate an inhibitory prelimbic cortex (PL)-dorsal raphe nucleus (DRN) circuit and that this circuit activation is required for the stress-buffering effects of ketamine. These data provide a basis for prophylactic use of ketamine in females.

## Introduction

Stress-related psychiatric disorders, such as depression and posttraumatic stress disorder (PTSD), affect females at nearly twice the rate of males (Kessler et al., 2005; Steiner et al., 2005) and are among the leading causes of disability worldwide (Kessler et al., 1995; Mathers et al., 2008). Only one third of patients prescribed conventional pharmacotherapies achieve full remission, underscoring the need for more effective therapeutic modalities. (Gaynes et al., 2009). Recently, it has been shown that a single subanesthetic dose (0.5 mg/kg, i.v.) of the nonselective NMDA receptor antagonist ketamine produces rapid and enduring therapeutic effects in individuals with treatment-resistant depression, anxiety, and PTSD (Berman et al., 2000; Zarate et al., 2006; Glue et al., 2017; Price et al., 2009; Feder et al., 2014). Accordingly, a growing body of research has been dedicated to identifying the underlying neurobiological mechanisms by which ketamine produces its effects.

Because of its clinical effectiveness, laboratory work has focused on two paradigms. In one, a single subanesthetic dose (10 mg/kg, i.p.) of ketamine is delivered at various time points before behavioral tests that are thought to reflect depressive or anxiety-related behavior. For example, ketamine delivered minutes to hours before behavioral testing prevents typical behavioral changes measured during the forced swim test (Garcia et al., 2008), tail suspension test (da Silva et al., 2010), novelty suppressed feeding test (NSF; Fuchikami et al., 2015) and the open-field test (Thelen et al., 2016). In the second, ketamine is given after exposure to a stressor to determine whether it would reverse stress effects on behavior. Ketamine delivered shortly after (0–24 h) exposure to a chronic unpredictable stressor reverses the effects of the stressor on NSF and sucrose preference (Li et al., 2011). Surprisingly, nearly all of the preclinical studies designed to identify the mechanistic actions of ketamine have focused on *male* rats. Indeed, a small number of studies have demonstrated differential sensitivity and responsivity of females and males to the direct and restorative effects of ketamine (Carrier and Kabbaj, 2013; Frnasceschelli et al., 2015; Zanos et al., 2016).

There has been a great deal of recent interest in factors that can lead to resilience in the face of adversity (for review, see Baratta et al., 2013), and interestingly, 3 recent reports indicate that single dose of ketamine can blunt the impact of stressors occurring as much as two weeks later (Amat et al., 2016; Brachman et al., 2016; McGowan et al., 2017). Unfortunately, none of these reports employed female subjects. Thus, we chose to explore the proactive effects of ketamine in female rats, as well as the underlying neural circuit-level processes that mediate such effects. We sought to determine whether ketamine delivered one week before an uncontrollable stressor (inescapable tailshock; IS) is sufficient to prevent anxiety-like behavior measured during juvenile social exploration (JSE) 24 h later, in a manner similar to that observed in male rats (Amat et al., 2016). IS-induced behavioral changes are mediated in part by activation of serotonergic (5-HT) neurons within the dorsal raphe nucleus (DRN; Maier and Watkins, 2005). Specifically, IS activates 5-HT neurons in the mid to caudal DRN (Grahn et al., 1999) leading to 5-HT release in projection regions that are proximal mediators of stress-induced behavioral changes, such as the basolateral amygdala (BLA; Amat et al., 1998; Christianson et al., 2010; Dolzani et al., 2016). Indeed, blockade of 5-HT2C receptors in the BLA eliminates the reduction in JSE produced by prior IS (Christianson et al., 2010). Therefore, we examined the effect of ketamine on IS-induced Fos activation in DRN 5-HT neurons to determine whether ketamine mitigates IS-induced DRN activation (Amat et al., 2016). Plastic changes in the prelimbic region (PL) of the medial prefrontal cortex (mPFC), a potent regulator of DRN activity (for review, see Maier and Watkins, 2010), are critical for the stress-buffering effects of ketamine (Li et al., 2010; Lepack et al., 2016; for review, see Duman and Krystal, 2016). Thus, we explored whether ketamine alters PL neural ensembles so that later IS now activates the same ensembles. Finally, we examined whether ketamine directly activates the PL-DRN pathway, and if so, whether PL-DRN pathway activation is critical for the protective effects of ketamine at the time of later IS.

## Materials and Methods

### Experimental design

The first set of experiments examined whether a single dose of ketamine would mitigate the behavioral and neurochemical effects of IS. Therefore, low-dose ketamine (10 mg/kg, i.p.), which is protective against stress outcomes in male rats (Li et al., 2010; Amat et al., 2016), was administered to female rats one week (7 d) before IS or HC treatment. Separate groups of rats received high-dose ketamine (40 mg/kg, i.p.), which is not implicated in stress resistance (Chowdhury et al., 2017), or saline. Anxiety-like behavior was assessed during a JSE test 24 h after the stressor. Thus, the experiment was a 2 (stress) × 3 (drug) factorial design. Two-way ANOVA was used for statistical analysis. Previous work performed using similar parameters (Dolzani et al., 2016) demonstrates that *n* = 9–12/group are sufficient to achieve statistical significance between groups. Three rats were considered statistical outliers (>2.5 SDs from the mean) and were excluded from the statistical analysis. The one-week time point was selected to dissociate the long-term stress-buffering effects of the ketamine from potential acute effects, which are not the focus of the present work. Additionally, previous work from our laboratory demonstrates that ketamine delivered one-week before IS in male rats protects against the typical effects of the stressor (Amat et al., 2016). DRN 5-HT activation was assessed in a separate group of rats using double label immunohistochemistry (IHC) to determine whether ketamine reduces overall stress-induced Fos expression within the DRN, and whether this reduction occurs in 5-HT neurons within the DRN. Fos and 5-HT expression were examined in the rostral, middle, and caudal DRN. Thus, the experiment was a 2 (stress) × 2 (drug) factorial design. Two-way ANOVA was used to separately analyze total 5-HT+ cells, total Fos+, and the percentage of 5-HT+ cells that also were also Fos+ between all possible groups. Previous work from our laboratory (Dolzani et al., 2016) demonstrates that *n* = 10–12/group (two brain slices per rat) are sufficient for detecting statistical differences in immunohistochemical labeling between groups. Four rats were considered statistical outliers (>2.5 SD from group mean for cell counts or insufficient staining for detection) and were removed from the analysis.

The major source of inhibitory control over DRN 5-HT neurons derives from descending PL glutamatergic pyramidal neurons that synapse preferentially on GABA interneurons in the DRN (Jankowski and Sesack, 2004). Interestingly, behavioral control blunts the impact of a stressor by activating this inhibitory pathway (Amat et al., 2005; Baratta et al., 2009). Moreover, the experience of control also has a prophylactic effect in that it blocks the behavioral effects of later uncontrollable stressors such as IS (Amat et al., 2006) and social defeat (Amat et al., 2010). After a prior experience with control, an uncontrollable stressor now does not activate DRN 5-HT neurons (Baratta et al., 2009). This occurs because the experience of control alters the PL-DRN pathway so that it is now activated by even uncontrollable stressors such as IS (Baratta et al., 2009). Thus, the second set of experiments sought to determine whether ketamine might engage the same mechanisms as behavioral control and alter PL neurons so that later IS now activates the PL. We used the recently developed immediate early gene (IEG) platform Robust Activity Marker (RAM) to interrogate neural activity at two time points: the time of initial ketamine injection (time 1), and the time of later IS (time 2). RAM provides a means for labeling neuronal ensembles activated by a particular temporally defined experience or event (for detailed explanation, see Sørensen et al., 2016). When combined with a subsequent IEG labeling technique, such as IHC, RAM allows for interrogation of neuronal activity at multiple time points. RAM utilizes a synthetic activity-dependent promoter (pRAM), which is driven by neuronal specific FOS and NPAS4 activity. pRAM activity drives expression of a tetracycline transactivator domain (tTA), which binds to a tetracycline-response element (TRE) and drives expression of the effector gene, mKate2. Temporal control over RAM is achieved using a modified Tet-Off system. Binding of the tTA to the TRE is inhibited in the presence of DOX (DOX+). In the absence of DOX (DOX-), effector gene transcription is enabled. Therefore, we used RAM to determine whether ketamine activates PL neurons at the time of injection (time 1) and whether a later experience with IS (time 2) now activates the same, or different, neuronal ensembles as did prior ketamine. Importantly, low- and high-dose ketamine were administered to separate groups of rats to determine whether IS-induced activation of neural ensembles previously activated by ketamine is specific to a dose of ketamine that protects against behavioral outcomes of stress. Thus, the experiment was a 2 (stress) × 3 (drug) factorial design. Two-way ANOVA was used to separately analyze total RAM, total Fos+, and the percentage of RAM+ that were also Fos+ between all groups. Previous work by Sørensen et al. (2016) demonstrates that *n* = 6–8/group (two brain slices per rat) are sufficient to detect statistical differences between groups in experiments performed using similar parameters. Five rats were considered outliers (>2.5 SDs from mean for cell counts or failure to detect expression of the injection control (eYFP).

The third experiment examined whether ketamine activates the PL-DRN circuit. Red fluorescent retrogradely transported microspheres (hereafter referred to as retrobeads; RBs) were injected into the DRN two weeks before rats receiving a single injection of ketamine. Two hours later, rats were killed and Fos expression was assessed in the PL and PL-DRN pathway. An independent samples *t* test was used to examine differences in total RB-positive (RB+) cells, total Fos+ cells, and the percentage of RB+ cells that were also Fos+; *n* = 6–8/group (two brain slices per rat) are sufficient to detect statistical differences between groups. Two rats were considered outliers (>2.5 SD from mean for cell counts) and excluded from analysis. Additionally, 4 rats were excluded from analysis due to inaccurate DRN RB injections.

The final experiment examined whether the PL-DRN circuit is necessary for the protective effects of ketamine. This requires selective inhibition of PL neurons that project to the DRN. Addressing this required the use of a dual viral intersectional genetic strategy to target Designer Receptors Exclusively Activated by Designer Drugs (DREADDs) to DRN-projecting PL neurons. A retrogradely transported AAV vector encoding Cre recombinase was delivered into the DRN and double-floxed AAV vector encoding the inhibitory DREADD receptor, (hM4Di) or mCherry (control virus), was delivered into the PL. This approach enables selective inhibition of neurons following systemic injection of clozapine-*N*-oxide (CNO; Armbruster et al., 2007; Ferguson et al., 2011; Rogan and Roth, 2011), as verified by reduced expression of *c-Fos* (Ferguson et al., 2011; Soumier and Sibille, 2014). to validate hM4Di-mediated inhibition of the PL-DRN pathway, we used an independent samples *t* test to assess total PL-DRN hM4Di/mCherry expression, total Fos+, and the percentage of hM4Di/mCherry expressing cells that were also Fos+. In a separate cohort, rats expressing hM4Di or mCherry in the PL-DRN pathway were injected with ketamine or saline, and one week later they received CNO injection 30 min before IS. Anxiety-like behavior was assessed 24 h later during JSE. Therefore, the experiment was a 2 (virus) × 2 (stress) × 2 (drug) factorial design. Three-way ANOVA was used for statistical analysis. Previous work using similar parameters (Dolzani et al., 2016) demonstrates that *n* = 10–12/group are sufficient to detect statistical significance. Three rats were considered statistical outliers (>2.5 SD from mean), and were removed from the statistical analysis. Additionally, two rats were excluded due to stress-related paw injuries, and six rats were excluded due to inaccurate or failed viral injections, as determined after completion of the experiment.

Data analysis for all one-way ANOVA, two-way ANOVA, and *t* tests was performed using Prism software (GraphPad). Three-way ANOVA was performed using Statview (SPSS). All experiments were performed using a between-subjects design and the effect of treatment was analyzed with unpaired *t* test (drug), one-way (stress), two-way (stress and drug), or three-way (stress and drug and virus) ANOVA. Main effects and interactions were considered statistically significant if *p* < 0.05. When appropriate, *post hoc* analyses and planned comparisons were performed using Tukey’s *post hoc* method. Values in graphs are represented as mean ± SEM.

### Rats

Adult female Sprague Dawley rats (250–300 g; Envigo) were pair housed on a 12/12 h light/dark cycle (lights on at 7 A.M. and off at 7 P.M.). Rats were housed with free access to food and water and were allowed to acclimate to colony conditions for 7 d before surgical or experimental procedures. All stereotactic surgeries were performed under 2.5% isoflurane (Piramal Critical Care) anesthesia. Rats received preoperative analgesic (meloxicam, 0.5 mg/kg, s.c.; Vetmedica) and antibiotic (Combi-Pen-48, 0.25 ml/kg, s.c.; Bimeda) before surgical procedures. Rats were given two weeks to recover from surgery before experimentation. All experiments were performed between 9 A.M. and 5 P.M. All animal procedures were approved by the Institutional Animal Care and Use Committee at the University of Colorado Boulder and conformed to National Institutes of Health Guidelines on the Care and Use of Laboratory Animals.

### Drug administration

In all experiments, ketamine (Ketalar, Pfizer) was administered at 10 or 40 mg/kg intraperitoneally. This dose was based on parameters previously described (Li et al., 2010; Amat et al., 2016; Chowdhury et al., 2017).

### Stress procedure

IS was delivered as previously described (Amat et al., 2005; Christianson et al., 2013). Briefly, rats were placed in a PlexiGlas restraint tube (8 × 18 cm, diameter × length). The rat’s tail was secured to a Plexiglas postprotruding from the rear portion of the box using medical tape and copper electrodes were placed around the tail. Shock was delivered to the rat’s tail with increasing intensity as the shock session progressed (33 trials at 1.0 mA, 33 trials at 1.3 mA, and 34 trials at 1.6 mA). Shock was delivered with an average intertrial interval (ITI) of 60 s. Rats were removed from the PlexiGlas boxes and placed in their home cage immediately after the last tailshock. Nonshocked homecage control (HC) rats were left undisturbed in the colony.

### JSE

JSE testing was conducted 24 h after IS or HC, as previously described (Amat et al., 2016). Any rats showing signs of injury (including injured hindpaws, forepaws, or toenails) after the stress session were excluded. Precautions are taken to minimize the frequency of these occurrences, but nonetheless injuries did occur in a small subset of subjects. Each experimental subject was singly assigned to an empty plastic cage with shaved wood bedding and a wire lid. Experimental subjects remained in the test cage for 45 min to 1 h before introducing a juvenile (28 ± 2 d old) female conspecific. An observer, blind to treatment, recorded exploratory behavior (allogrooming, licking, sniffing, and pinning) initiated by the experimental subject during a 3-min test. JSE test scores were reported as a total time (s) of social exploration during the 3-min test.

### Fluorescent IHC

Staining for Fos was performed using a general immunofluorescence protocol. Following a series of washes in 0.01 M PBS containing 0.5% Triton X-100, slices were incubated overnight in a PBS blocking solution containing 0.5% Triton X-100 (PBST) and 2.5% bovine serum albumin (BSA) at 4°C. Then, slices were washed in PBST and incubated for 24 h at room temperature (RT) in rabbit polyclonal primary antibody (1:1000; Santa Cruz Biotechnology) in blocking solution. After a series of PBS washes, slices were incubated for 2 h at RT in Alexa Fluor 405 goat antirabbit secondary antibody (1:250; Life Technologies). After a series of PBS washes, tissue was floated onto slide glass and coverslipped with Vecta Shield (Vector Labs).

### Image analysis for fluorescent IHC experiments

Brain sections were observed using a Nikon N-SIM structured illumination super-resolution laser scanning confocal microscope (Nikon). Images were captured using NIS Elements (Nikon) and analyzed using FIJI (ImageJ). All digital images were captured using a 20× objective. For imaging and quantification of PL (taken between AP: +2.5 mm to +3.0 mm relative to bregma) boundaries were based on those previously described (Baratta et al., 2009) with consultation to the brain atlas (Paxinos and Watson, 2011). In the PL-DRN retrograde tracing experiment, RB+ cell bodies in the PL were observed using a 546-nm laser line and were pseudocolored red. Fos+ nuclei were observed using a 405-nm laser line and were pseudocolored blue. RB+ and Fos+ cells in the PL were quantified and recorded separately. Colocalization of RB and Fos was reported when a red/magenta cell body representing the intermixture of the two fluorophores was observed and verified to be overlapping RB+ and Fos+ cells. In the PL RAM experiment, eYFP-labeled neurons (site of viral injection) were observed using a 488-nm laser line and were pseudocolored green. eYFP expression was confirmed in all subjects included in statistical analysis. Following confirmation of accurate viral injection, RAM-labeled nuclei were observed using a 546-nm laser line and were pseudocolored red. Fos+ nuclei were observed using a 405-nm laser line and were pseudocolored blue. RAM+ and Fos+ cells in the PL were quantified and recorded separately. Colocalization of RAM and Fos was reported when a magenta nucleus representing the intermixture of the two fluorophores was observed and verified to be overlapping RAM and Fos. In the DREADD-mediated PL-DRN pathway silencing experiment, DREADD or mCherry positive cells were observed using a 546-nm laser line and were pseudocolored red. Fos+ cells were observed using a 405-nm laser line and were pseudocolored green. DREADD+ and Fos+ cells in the PL were quantified and recorded separately. Colocalization of DREADD and Fos was reported when a yellow nucleus representing the intermixture of the two fluorophores was observed and verified to be overlapping DREADD and Fos. For each subject, two separate counts were taken from PL tissue spanning AP: +2.5 mm to +3.0 mm. The two counts for each subregion were averaged and used for statistical analysis.

### Effect of ketamine on later JSE

Rats randomly assigned to drug and stress treatment received a single systemic injection of ketamine (10 or 40 mg/kg, i.p.) or vehicle one week before IS or HC. Twenty-four hours after the completion of IS or HC, rats received JSE testing.

### Effect of ketamine on stress-induced Fos activation in the DRN

#### Tissue preparation

Rats injected with ketamine 1 w before IS or HC were deeply anesthetized with sodium pentobarbital (65 mg/kg) 2 h after the last tailshock or at the same time for rats assigned to HC. Rats were transcardially perfused with 100-ml ice-cold 0.9% saline, immediately followed by 250-ml 4% paraformaldehyde in 0.1 M phosphate buffer (PB; pH ∼7.4). Brains were postfixed overnight in the same fixative and transferred to a 30% sucrose solution in 0.1 M PB then stored at 4°C until sectioning. Coronal brain sections containing DRN were obtained at 35 μm. DRN tissue used for IHC was placed directly into a 24-well plate.

#### IHC for Fos and 5-HT

Staining for Fos and 5-HT was conducted as previously described (Grahn et al., 1999). Staining for Fos was conducted using the avidin-biotin-horseradish peroxidase (ABC) method. Following a series of washes in 0.1 M PBS, sections were incubated in a 0.9% hydrogen peroxide solution to quench endogenous peroxidases. Then, sections were incubated for 24 h at RT with Fos primary antibody (1:15,000; Santa Cruz Biotechnology) in a blocking solution containing 2% normal goat serum (NGS), 0.5% Triton X-100 and 0.1% sodium azide. Following the primary antibody incubation, sections were incubated for 2 h at RT in biotinylated goat anti-rabbit secondary antibody (1:200; Jackson ImmunoResearch) in blocking solution. After a series of PBS washes, slices were then incubated in ABC for 1 h at RT. Next, sections were washed in 0.1 m PB and then exposed to a solution containing 3,3-diaminobenzidine (DAB), cobalt chloride, nickel ammonium sulfate, ammonium chloride and glucose oxidase in PB. The peroxidase reaction was initiated by the addition of a glucose solution that reacted with the tissue for ∼7–10 min. The reaction was terminated by washing sections with PBS. Tissue was floated onto slide glass and cover slipped for later analysis. 5-HT staining was conducted using the peroxidase anti-peroxidase (PAP) method. Following a series of washes in PBS, excess background 5-HT staining was prevented by incubating sections in blocking solution for 0.5 h. Next, tissue was incubated in blocking solution of 5-HT antibody (rabbit polyclonal 1:10,000; Jackson ImmunoResearch) for 48 h at RT. Goat anti-rabbit secondary antibody (1:200; Jackson ImmunoResearch) was applied to the tissue for 2 h after a series of PBS washes. This step was followed by another series of PBS washes and incubation with PAP antibody (1:200; Jackson ImmunoResearch) for 2 h. Following a series of washes in PBS, tissue was incubated in a solution containing DAB and glucose oxidase. The peroxidase reaction was initiated by addition of glucose and continued for 15 min. After a final series of PBS washes, tissue was mounted on slides and allowed to dry overnight. Slides were coverslipped with Permount.

#### Image analysis

Brain sections were observed using a bright field microscope (Olympus BX-61, Olympus America) and analyzed using cellSens software (Olympus America). All digital images were captured using a 20× objective. Images of DRN were taken using parameters similar to those previously described (Grahn et al., 1999). Sections corresponding to an AP coordinate of -1.36, -1.00, and -0.70 mm relative to interaural zero were taken for rostral, middle, and caudal DRN, respectively. Fos-positive nuclei in each subregion of the DRN were observed as dark brown or black round/ovoid spots. 5-HT-stained cell bodies were observed as light brown particles with and without unstained nuclei. Colocalization of Fos and 5-HT was observed as a light brown cell body with a black stained nucleus. For each subject, two separate counts were taken from different slices within each subregion of the DRN. The two counts for each subregion were averaged and used for statistical analysis.

### Effect of ketamine on activation of the PL-DRN pathway

#### Microinjection of retrograde tracer

A small window (1 × 1 mm) was drilled into the skull and red fluorescent RBs (Lumafluor) were microinjected into the DRN (AP: -8.0 mm relative to bregma, DV: -6.7 mm from skull surface, ML: ±0.0 mm relative to midline) using a 10-μl Hamilton syringe and a 31-gauge metal needle with a 45° beveled tip. The total injection volume (0.3 μl) and flow rate (0.1 μl/min) was controlled with a microinjection pump (UMP3-1; World Precision Instruments). This injection volume was chosen based on pilot experiments in which robust RB expression was observed within the PL two weeks after 0.3-μl injection, while maintaining localized injections. Following injection, the needle was left in place for an additional 10 min to allow for RB diffusion, after which the needle was withdrawn. The small scalp incision was closed using Vetbond (3M). Preoperative antibiotic (Combi-Pen-48, 0.25 ml/kg, s.c.) and a postoperative analgesic (meloxicam, 2 mg/kg, s.c.) was administered to all rats. Two weeks after RB microinjection, rats received a systemic injection of ketamine or saline and were killed 2 h later. RB microinjections were considered successful if expression was visibly confined to DRN in coronal sections of brain obtained after completion of the experiment. Only rats with accurate RB injections were used for statistical analysis.

#### Tissue preparation

Rats were deeply anesthetized with sodium pentobarbital (65 mg/kg) at 2 h following the ketamine or saline injection. Rats were transcardially perfused with 100-ml ice-cold 0.9% saline, immediately followed by 250-ml 4% paraformaldehyde in 0.1 M PB (pH ∼7.4). Brains were postfixed overnight in the same fixative and transferred to a 30% sucrose solution in 0.1 M PB then stored at 4°C until sectioning. Coronal brain sections containing PL were obtained at 35 μm. PL tissue used for IHC was placed directly into a 24-well plate. DRN tissue was mounted onto glass slides and coverslipped with VectaShield (Vector Labs) mounting medium.

### Effect of ketamine on PL neural ensembles at the time of initial injection and at the time of later stress

#### Virus

Adeno-associated virus (AAV) vectors were used to target the RAM-NLS-mKate2 (RAM) fusion transgene to neurons. RAM cassettes were packaged in AAV vectors serotyped with AAV1 coat proteins (titers: 2.18 × 10^13^ genome copies/ml) by Virovek. hSYN-eYFP (eYFP) cassettes packaged in AAV vectors serotyped with AAV1 coat proteins were (titers: 3.86 × 10^12^ genome copies/ml) by University of Pennsylvania. Before injection, RAM was mixed with eYFP (8.5-μl RAM/1.5-μl eYFP). eYFP is expressed in the absence of neuronal activation, and serves as method of determining injection accuracy following completion of the experiment. This ensures that in the absence of RAM expression, injection verification can be achieved.

#### Viral vector delivery

Rats were placed on doxycycline chow (DOX; 200 mg/kg, BioServ) 24 h before surgery as previously described (Sørensen et al., 2016). On the day of surgery, rats were anesthetized and a single unilateral injection of RAM was directed to the PL (AP: +2.5 relative to bregma, DV: -2.0 relative to pial surface, ML: ±1.0 mm) using injection parameters similar to those described above. 1000 nl of RAM was delivered at a rate of 100 nl/min. Following completion, the injection needle was left in place for an additional 10 min to allow for diffusion of virus, after which the needle was withdrawn. Postoperative care was performed as described above.

#### Labeling of neuronal ensembles at the time of ketamine injection and later stress

Following injection of RAM, rats remained on DOX chow (200 mg/kg) for 96 h. This period of time is sufficient to prevent basal induction of RAM in the absence of a salient event, such as drug injection. DOX chow was withdrawn and replaced with standard lab chow 96 h later. Removing DOX chow <96 h prevents RAM expression due to circulating DOX-mediated transgene suppression (data not shown). Rats remained undisturbed in the colony for 96 h following the withdrawal of DOX. Following the 96 h DOX- interval, rats randomly assigned to drug and stress treatment were injected with either low-dose ketamine (10 mg/kg, i.p.), high-dose ketamine (40 mg/kg, i.p.), or saline. Twenty-four hours later, rats were placed back on DOX chow (1000 mg/kg). This time interval allows for robust expression of RAM, while minimizing nonspecific RAM expression. 48 h later, rats received IS or HC and were perfused 2 h later.

#### Tissue preparation

Rats were deeply anesthetized and perfused as described above. Coronal brain sections containing PL were obtained at 35 μm. PL tissue used for IHC was placed directly into a 24-well plate. Fluorescent IHC was performed as described above.

### Effect of PL-DRN inhibition on the stress buffering effects of ketamine

#### Virus

AAV2-retro-eSyn-eGF-T2A-iCre-WPRE (Cre) cassettes were packaged in AAV vectors serotyped with AAV coat proteins (titers: genome copies/ml) by Vector Biolabs. AAV8-hSyn-DIO-hM4Di(G_i_)-mCherry (hM4Di) cassettes were packaged in AAV vectors serotyped with AAV8 coat proteins (titers: 4.3 × 10^12^ genome copies/ml) by Addgene. AAV-hSyn-DIO-mCherry (mCherry) cassettes were packaged in AAV vectors serotyped with AAV5 coat proteins were (titers: 2.1× 10^13^ genome copies/ml) by Addgene.

#### Viral vector delivery

A small window (1 × 1 mm) was drilled into the skull and Cre was microinjected into the DRN (AP: -8.0 mm relative to bregma, DV: -6.7 mm from skull surface, ML: ±0.0 mm relative to midline) using a 10-μl Hamilton syringe and a 31-gauge metal needle with a 45° beveled tip. The total injection volume (1 μl) and flow rate (0.1 μl/min) was controlled with a microinjection pump (UMP3-1; World Precision Instruments). Following injection, the needle was left in place for an additional 10 min to allow for virus diffusion, after which the needle was withdrawn. The small scalp incision was closed using Vetbond (3M). Preoperative antibiotic (Combi-Pen-48, 0.25 ml/kg, s.c.) and a postoperative analgesic (meloxicam, 2 mg/kg, s.c.) was administered to all rats. Five days later using identical surgical procedures, hM4Di or mCherry was microinjected into the PL (AP: +2.5 mm relative to bregma, DV: -2.0 mm from pial surface, ML: ±0.5 mm relative to midline).

#### hM4Di validation

To determine whether CNO delivery is sufficient to prevent activation of PL-DRN neurons, rats targeted with hM4Di to the PL-DRN pathway received a single injection of CNO (3.0 mg/kg, i.p.) or vehicle 30 min before receiving a single injection of ketamine (10 mg/kg, i.p.). Rats were perfused 90 min later. Coronal brain sections containing PL were obtained at 35 μm. PL tissue used for IHC was placed directly into a 24-well plate. Fluorescent IHC was performed and Fos expression was examined in the PL-DRN pathway. Only rats with accurate bilateral expression of hM4Di were included in the statistical analysis.

#### Estrous cycle determination

Vaginal lavage was performed before stress treatment. A blunt-tipped eyedropper filled with a small amount of 0.9% sterile saline was inserted into the vagina. Fluid was quickly expelled two to three times to gently wash off and collect vaginal cells (∼0.25 ml). A drop was placed onto a glass slide and immediately examined with a 40× objective lens. Characteristic changes in the cytological appearance of the samples were used to identify the cycle stage: diestrus I/II (presence of nucleated cells and leucocytes), proestrus (presence of nucleated cells), and estrus (presence of anucleated squamous cells).

#### PL-DRN silencing

Rats targeted with hM4Di or mCherry to the PL-DRN pathway were randomly assigned to drug and stress treatment. Rats received a single systemic injection of ketamine or vehicle one-week before IS or HC. Rats received a single injection of CNO (3.0 mg/kg, i.p.) 30 min before IS or HC treatment. Twenty-four hours after the completion of IS or HC, behavior was assessed during JSE. Following completion of behavioral testing, rats were deeply anesthetized and perfused using saline and 4% paraformaldehyde. Coronal sections containing PL and DRN were mounted directly onto slides and accurate viral expression in the PL-DRN pathway was confirmed. Only rats with accurate bilateral expression of hM4Di or mCherry were included in the statistical analysis.

#### Statistics

Data analysis was performed with Prism software (GraphPad). The effect of treatment was analyzed with unpaired *t* test (drug), or one-way (stress), two-way (stress and drug), or three-way (stress and drug and virus) ANOVA. Main effects and interactions were considered statistically significant if *p* < 0.05. When appropriate, *post hoc* analyses and planned comparisons were performed using Tukey’s *post hoc* method. Values in graphs are represented as mean ± SEM. The results of all statistical analyses are listed in Statistics.

## Results

### Effect of systemic ketamine on JSE and DRN 5-HT activation

#### Behavior

**Figure 1.**
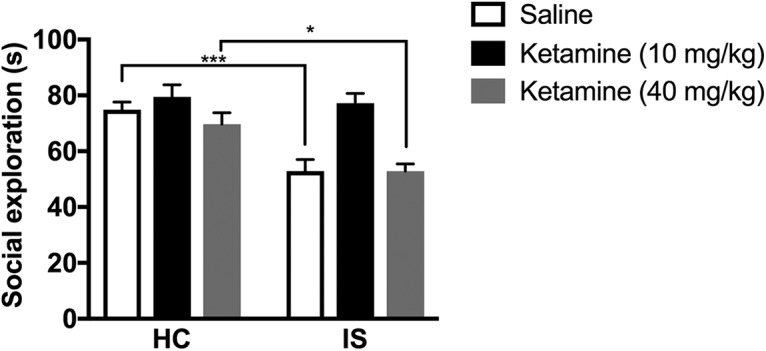
Low-dose ketamine protects female rats against later stress-induced JSE deficits. ***A***, Rats received low (10 mg/kg, i.p.) or high (40 mg/kg, i.p.) dose ketamine one week before stress or HC treatment. JSE was measured 24 h after stress or HC treatment. Tukey’s *post hoc* method: *p < 0.05, ***p < 0.001. Bars represent group mean ± SEM.

Rats received either low-dose ketamine (10 mg/kg, i.p.), high-dose ketamine (40 mg/kg, i.p.) or saline (i.p.) 7 d before IS or HC (*n* = 9–10/group). Anxiety-like behavior was assessed during JSE 24 h later. Figure 1 shows the total time spent interacting during the 3-min JSE test. Prior low- or high-dose ketamine delivered to HC, rats had no effect on JSE, relative to saline injected HC controls. JSE was significantly reduced in rats previously injected with saline or high-dose ketamine that received IS. Importantly, low-dose ketamine delivered one week prior completely blocked the effect of IS on JSE. Two-way ANOVA revealed significant main effects of stress (*F*_(1,52)_ = 21.13, *p* < 0.0001), drug (*F*_(2,52)_ = 12.62, *p <* 0.0001), and a stress by drug interaction (*F*_(2,52)_ = 3.97, *p* = 0.025). Tukey’s *post hoc* method revealed that rats administered saline or high-dose ketamine before IS were indistinguishable from each other but differed from all other groups (*p* < 0.05). Rats administered low-dose ketamine and IS differed significantly from rats that received saline and IS or high-dose ketamine and IS, while IS rats previously administered low-dose ketamine were indistinguishable from HC rats that previously received saline, low-dose ketamine, or high-dose ketamine. Social exploration was significantly reduced in rats administered saline and IS (*p* = 0.0009) or high-dose ketamine and IS (*p* = 0.03) compared to their respective home cage group.

#### DRN 5-HT activation

**Figure 2.**
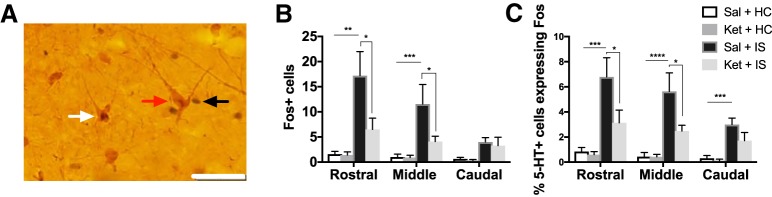
Low-dose ketamine blunts stress-induced DRN activation. ***A***, Representative photomicrograph showing a 5-HT+ cell (red arrow), Fos+ cell (black arrow), and double-labeled cell expressing 5-HT and Fos (white arrow). ***B***, Total number of Fos+ cells (left) and percentage of 5-HT+ cells also expressing Fos within the rostral, middle, and caudal subregions of the DRN. Tukey’s *post hoc* method: *p < 0.05, **p < 0.01, ***p < 0.001, ****p < 0.0001. Bars represent group mean ± SEM.

To determine whether ketamine prevents typical IS-induced activation of the DRN, we assessed Fos immunoreactivity (Fos+), 5-HT immunoreactivity (5-HT+), and the percentage of 5-HT cells also expressing Fos (%5-HT+ cells expressing Fos) in the rostral, middle, and caudal DRN of rats injected one week prior with low-dose ketamine or vehicle (Fig. 2). We focused specifically on these three subregions of the DRN because prior work demonstrates that, in male rats, IS activates the middle and caudal DRN (Grahn et al., 1999). Similarly, the 2-h time point was chosen based on previous experiments demonstrating stress-induced Fos in the DRN (Grahn et al., 1999). Figure 2*A* shows a representative photomicrograph denoting a 5-HT+ cell, a Fos+ cell, and a 5-HT+ cell also expressing Fos. Two-way ANOVA was used to examine whether the total number of 5-HT+ cells varied between groups within the rostral, middle, or caudal DRN. No differences in the total number of 5-HT+ cells were observed (*p* > 0.05) between stress groups for the rostral, middle, or caudal subregions of the DRN (data not shown). Fos+ cells were examined within each DRN subregion for the different treatment groups (Fig. 2*B*, left). Within the rostral DRN, a two-way ANOVA revealed a main effect of drug (*F*_(1,38)_ = 4.29, *p* = 0.046) and a main effect of stress (*F*_(1,38)_ = 15.22, *p* = 0.0004). Tukey’s *post hoc* analysis revealed that IS rats show an increase in Fos+, relative to rats that received HC (*p* < 0.05). Fos+ was significantly reduced in rats previously administered ketamine that received IS compared to rats administered saline before IS (*p* < 0.05). Within the middle DRN, stress increased Fos+; however, this effect was partially prevented by prior ketamine. These observations were confirmed by two-way ANOVA, which revealed main effects of stress (*F*_(1,39)_ = 14.94, *p* = 0.0004), drug (*F*_(1,39)_ = 4.539, *p* = 0.04), and a stress × drug interaction (*F*_(1,39)_ = 4.303, *p* = 0.045). *Post hoc* analysis demonstrated that Fos+ was significantly increased in IS rats, relative to HC rats (*p* < 0.01). Ketamine delivered before IS reduced Fos+ relative to IS rats that received saline (*p* < 0.05). The number of Fos+ cells in the caudal DRN was greater after IS than HC. This was confirmed by two-way ANOVA, which revealed a main effects of stress (*F*_(1,36)_ = 9.446, *p* = 0.004). Not surprisingly, IS produced a robust increase in the number of 5-HT+ cells (5-HT+ Fos) in the rostral, middle and caudal DRN (Fig. 2*B*, right). Within the rostral DRN, a two-way ANOVA revealed main effects of stress (*F*_(1,38)_ = 20, *p* < 0.0001) and drug (*F*_(1,38)_ = 4.255, *p* = 0.046). Compared to HC rats previously administered saline, IS rats that received saline showed enhanced 5-HT+ cells also expressing Fos (*p* < 0.05). The number of 5-HT+ cells also expressing Fos was reduced in IS rats that previously received ketamine compared to IS rats that received saline (*p* < 0.05). Within the middle DRN, two-way ANOVA yielded main effects of stress (*F*_(1,39)_ = 26.58, *p* < 0.0001), drug (*F*_(1,39)_ = 5.048, *p* = 0.03), and a stress × drug interaction (*F*_(1,39)_ = 5.037, *p* = 0.03). As observed in the rostral DRN, rats previously administered saline or ketamine that received IS showed an enhancement in the number of 5-HT+ cells also expressing Fos, compared to rats that received saline or ketamine and HC (*p* < 0.0001). Prior ketamine reduced the IS-induced enhancement of 5-HT+ cells also expressing Fos, relative to saline treated rats that received IS (*p* < 0.05). Finally, within the caudal DRN, two-way ANOVA revealed a main effect of stress (*F*_(1,36)_ = 23.01, *p* < 0.0001). Saline treated rats that received IS showed enhanced 5-HT+ cells also expressing Fos compared to saline and ketamine treated HC rats (*p* < 0.001). In sum, IS activated DRN 5-HT neurons, and this activation was substantially blunted by ketamine administered 7 d earlier.

#### Effect of ketamine on the activity of PL at the time of stress

**Figure 3.**
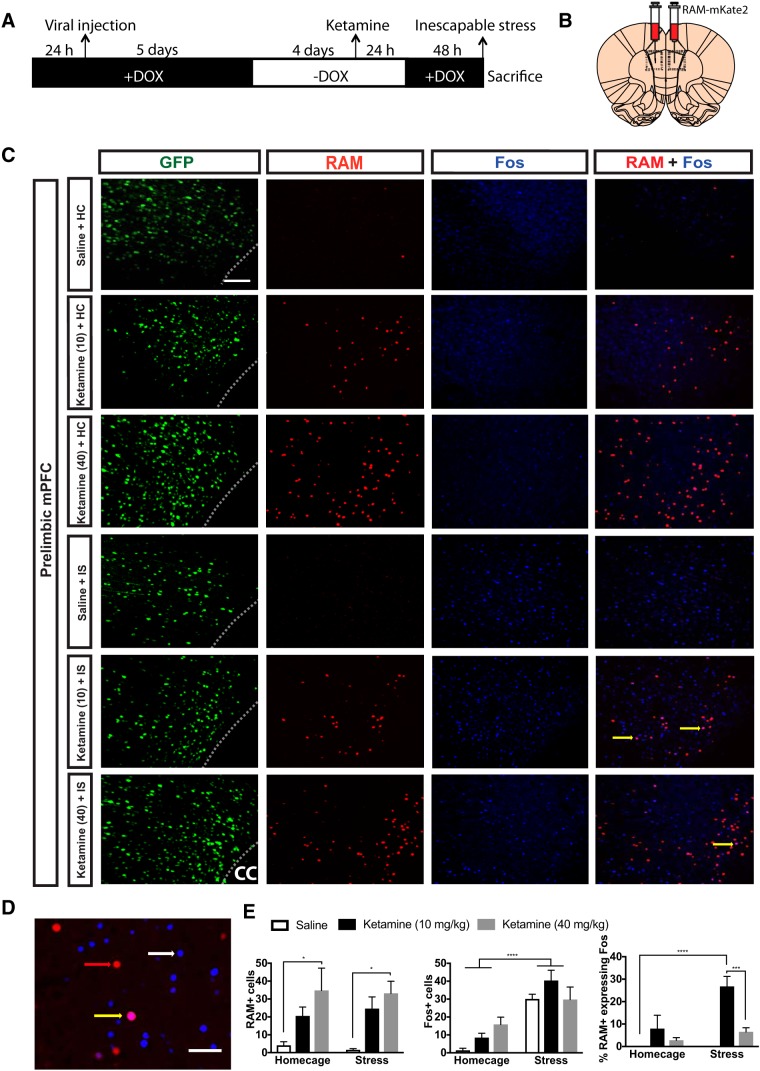
Ketamine-induced RAM labeling of a transcriptionally active neural ensemble that is later activated by uncontrollable stress. ***A***, Schematic timeline of the experimental procedure. Rats were injected with AAV-NLS-RAM-mKate2 (RAM). Nine days later, rats received a single systemic injection of low-dose ketamine (10 mg/kg, i.p.), high-dose ketamine (40 mg/kg, i.p.), or saline. Seventy-two hours later, rats were subjected to IS or left undisturbed in their homecage. ***B***, Schematic diagram of a coronal section of rat brain demonstrating the location of RAM injections into the PL. Viral injection verification was confirmed with eYFP and RAM + Fos were quantified in the PL subregion denoted with a dashed rectangle. ***C***, Representative images of the PL showing eYFP+ cells (green), RAM+ cells (red), Fos+ cells (blue), and RAM cells expressing Fos (denoted with yellow arrows in far right panel). Scale bar: 100 μm and applies to all images. ***D***, Enlarged image of the PL showing RAM (denoted with red arrow), Fos (denoted with white arrow), and a RAM cell expressing Fos (denoted with yellow arrow). Scale bar: 50 μm. ***E***, Number of RAM labeled cells (left), Fos+ cells (middle), and percentage of double-labeled RAM+ cells that also express Fos (right) in the PL of rats that received ketamine or saline followed by later IS or HC treatment. Tukey’s *post hoc* method: *p < 0.05, **p < 0.01, ***p < 0.001 for graph of RAM+ cells and % RAM cells expressing Fos. Two-way ANOVA main effect: ***p < 0.001 for graph of Fos+. Bars represent group mean ± SEM.

Previous work demonstrates a role for deep layer (Layer V/VI) PL neurons in the stress-buffering effects of both ketamine and behavioral control (Amat et al., 2005, 2016; Varela et al., 2012). Therefore, we sought to determine whether prior ketamine might induce activation of neural ensembles within the PL, so that a later experience with IS now would activate the PL, in a manner consistent with behavioral control in males (Baratta et al., 2009). Such findings would suggest that the protective effects of ketamine are mediated, at least in part, by enduring changes in a neural node commonly studied with regard to inhibitory control over the DRN (Amat et al., 2005; Baratta et al., 2009). Figure 3*A* shows a schematic diagram of the experimental timeline. First, we sought to determine whether a single injection of ketamine (Fig. 3*B*) would induce RAM (RAM+) expression, indicative of PL activation (Fig. 3*C*,*D*). To optimize activity-dependent RAM expression, we assessed two different time intervals between the removal of DOX and injection of ketamine. Using this approach, we found that rats should be off DOX (DOX-) for 96 h before ketamine injection to observe RAM labeling. Worth noting, rats taken off DOX 48 h before ketamine showed no change in RAM, relative to saline treated rats (*p* = 0.65), suggesting that DOX was still present 48 h after DOX removal. High-dose ketamine, but not low-dose ketamine or saline, induced robust expression of RAM+ in the PL (main effect of drug, *F*_(2,31)_ = 10.71, *p* = 0.0003). This was not altered by later IS, as would be expected since the animals were placed back on DOX 24 h after receiving an injection of ketamine. *Post hoc* analysis revealed that rats previously injected with high-dose ketamine who later received HC showed enhanced RAM+ expression relative to saline injected rats that later received HC (*p* = 0.039). Similarly, rats previously injected with high-dose ketamine who later received IS showed enhanced RAM+ expression relative to saline injected rats that later received ID (*p* = 0.023). Next, total Fos+ was examined following IS (Fig. 3*C*,*D*). As previously described (Baratta et al., 2009), IS increased the number of Fos+ cells in the PL. ANOVA revealed main effects of stress (*F*_(1,31)_ = 43.69, *p* < 0.0001). Rats previously injected with saline that received later IS showed enhanced Fos+ in the PL, relative to rats previously injected with saline that received HC (*p* = 0.002). Similarly, rats previously injected with low-dose ketamine that received later IS showed enhanced Fos+, relative to rats injected with low-dose ketamine that received HC (*p* = 0.0004). Finally, the percentage of RAM+ cells also expressing Fos was examined to determine whether ketamine-induced experiential ensembles are recruited at the time of later IS (Fig. 3*C*,*D*). ANOVA yielded main effects of stress (*F*_(1,31)_ = 8.55, *p* = 0.0064), drug (*F*_(2,31)_ = 16.31, *p* < 0.0001), and a stress × drug interaction (*F*_(2,31)_ = 5.031, *p* = 0.013). Strikingly, IS produced a profound increase in Fos+ in RAM+ PL neurons previously activated by low-dose, but not high-dose, ketamine, compared to rats that received HC and prior saline (*p* < 0.0001) and IS and prior high-dose ketamine (*p* < 0.001).

#### Effect of ketamine on activity of the PL-DRN pathway

**Figure 4.**
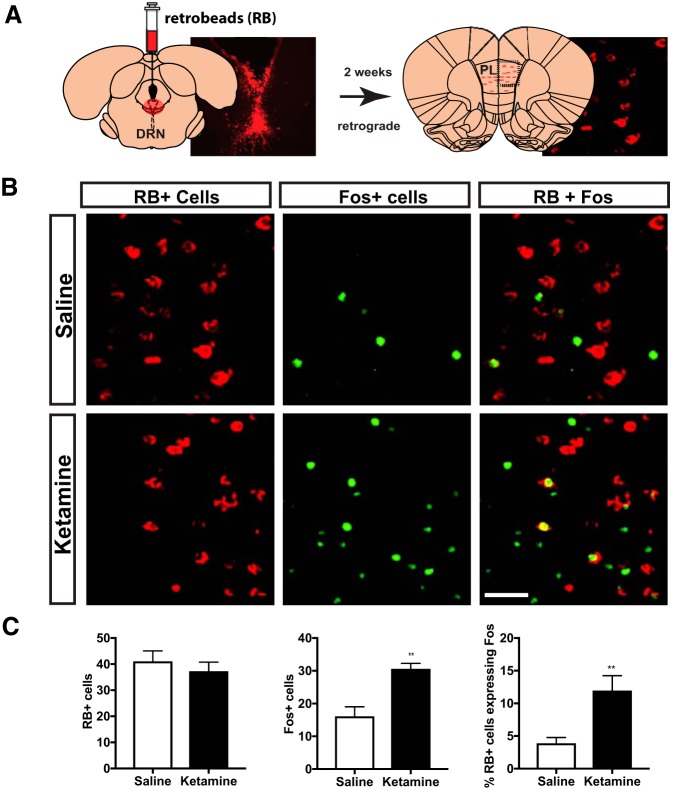
Acute ketamine activates the PL-DRN pathway. ***A***, Schematic diagram of the experimental procedure. Rats were injected with red fluorescent RBs in the DRN. Two weeks later, rats received a single systemic injection of ketamine (10 mg/kg, i.p.) or saline. Two hours later, rats were killed. RB and Fos expression was assessed in the PL. ***B***, Representative images of the PL showing RB+ cells (red), Fos+ cells (green), and RB + Fos (denoted with white arrows in far right panel). Scale bar: 50 μm and applies to all images. ***C***, Number of RB labeled cells (left), Fos+ cells (middle), and percentage of double-labeled RB-labeled cells that also express Fos (right) in the PL of rats that received ketamine or saline. Unpaired *t* test: **p < 0.01. Bars represent group mean ± SEM.

Previous work from our laboratory has demonstrated that the PL-DRN pathway is critically involved in the stress-buffering effects of behavioral control, which shares several common actions with proactive ketamine. (Amat et al., 2005, 2014, 2016; Baratta et al., 2009). The present experiment sought to establish whether prior ketamine might activate the PL-DRN pathway in a manner similar to that described by Baratta et al. (2009) for behavioral control. Figure 4*A* shows a schematic diagram of the injection procedure. Fos expression was examined in retrogradely (RB+) labeled PL-DRN neurons (Fig. 4*B*). As previously described, RB injections in the DRN yielded expression confined primarily to the deep Layers (V/VI) of PL and IL, with some expression in the dorsal anterior cingulate (Gabbott et al., 2005; Baratta et al., 2009; Gonçalves et al., 2009; Amat et al., 2016). Worth noting, the total number of RB+ PL neurons did not differ between groups (*p* = 0.496; Fig. 4*C*, left). To determine whether ketamine activates the PL, we analyzed the total number of Fos+ cells in rats that received ketamine or saline (*n* = 8 and *n* = 7, respectively; Fig. 4*C*, middle). A single injection of ketamine increased the number of Fos+ cells in the PL (*t*_(13)_ = 4.145, *p* = 0.001). Next, we assessed whether ketamine activates the PL-DRN by quantifying the percentage of DRN-projecting PL neurons expressing Fos (Fig. 4*C*, right). Indeed, ketamine produced a robust increase in activation of the PL-DRN pathway (*t*_(13)_ = 3.453, *p* = 0.004).

#### Effect of DREADD-mediated silencing of the PL-DRN pathway on the stress-buffering effects of ketamine

**Figure 5.**
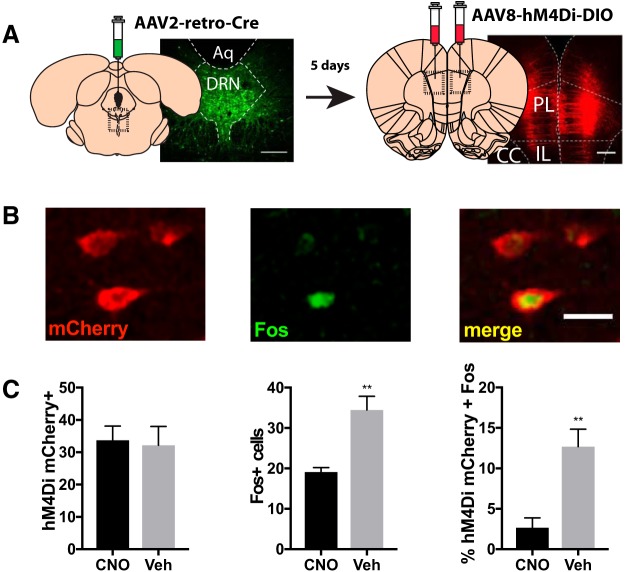
hM4Di prevents the effects of prior ketamine on activation of the PL-DRN pathway. ***A***, Schematic diagram of the injection procedure. Rats were injected with retrogradely transported AAV vectors encoding Cre and eGFP. Five days later, rats were injected with Cre-dependent AAV vectors encoding hM4Di-mCherry Scale bar: 250 μm and applies to both images. ***B***, Representative images of the PL showing hM4Di-mCherry expression (left), Fos expression (middle), and the colocalization of hM4Di-mCherry and Fos (right). Scale bar: 50 μm. ***C***, Number of hM4Di-mCherry labeled cells (left), Fos+ cells (middle), and percentage of hM4Di-mCherry expressing cells that also express Fos (right) in the PL of rats that received ketamine before CNO or vehicle. Unpaired *t* test: **p < 0.01. Bars represent group mean ± SEM.

The goal of the present experiment was to assess whether chemogenetic inactivation of the PL-DRN pathway at the time of IS would mitigate the protective effects of prior ketamine. First, the ability of hM4Di to suppress ketamine-mediated PL-DRN pathway activation was validated (Fig. 5). Figure 5*A* depicts the injection procedure used to target hM4Di to the PL-DRN pathway. Representative images of hM4Di, Fos+, and a hM4Di-positive cells also expressing Fos are shown in Figure 5*B*. The total number of mCherry-expressing cells in the PL was quantified (Fig. 5*C*, left). As previously shown, DRN-projecting PL neurons were primarily localized to the deep layers of PL (Gabbott et al., 2005; Baratta et al., 2009; Gonçalves et al., 2009). The total number of PL-DRN hM4Di labeled cells did not differ between the two groups (*t*_(8)_ = 0.22, *p* = 0.83; Fig. 5*C*, left). In agreement with the results of the PL-DRN retrograde tracing experiment, ketamine significantly increased the number of Fos+ cells in the PL (Fig. 5*C*, middle). Compared to rats injected with vehicle, CNO significantly reduced ketamine-induced increases in PL Fos+ (*t*_(8)_ = 4.337, *p* = 0.003). Next, Fos+ confined to the PL-DRN pathway was examined (Fig. 5*C*, right). Rats injected with CNO showed significantly lower ketamine-induced PL-DRN Fos+ compared to rats injected with saline (*t*_(8)_ = 4.051, *p* = 0.004).

**Figure 6.**
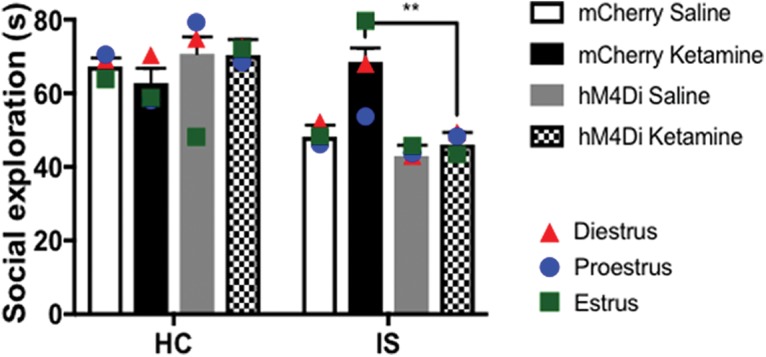
hM4Di-mediated inhibition of the PL-DRN pathway prevents the prophylactic effect of prior ketamine on JSE. Rats previously injected with hM4Di or mCherry targeted to the PL-DRN pathway received a single injection of saline or ketamine one week before IS or HC. JSE was assessed 24 h after IS or HC. Tukey’s *post hoc* method: **p < 0.01. Bars represent group mean ± SEM. Symbols represent mean social exploration for rats that received HC or IS during diestrus, proestrus, or estrus.

In a separate cohort of rats expressing PL-DRN hM4Di or mCherry, ketamine or saline was administered one week before IS or HC. Thirty minutes before IS or HC, all rats were injected with CNO (3.0 mg/kg, i.p.). Therefore, the experiment was a 2 (stress) × 2 (drug) × 2 (virus) factorial design. Twenty-four hours after IS or HC, rats received JSE testing. Figure 6 shows the time spent interacting during the JSE test. Three-way ANOVA revealed a main effect of stress (*F*_(_*_1_*_,83)_ = 46.98, *p* < 0.0001), a stress × drug interaction (*F*_(1,83)_ = 8.81, *p* = 0.004), a stress × virus interaction (*F*_(1,83)_ = 16.31, *p* = 0.0001), and a stress × drug × virus interaction (*F*_(1,83)_ = 9.97, *p* = 0.023). Within the HC groups, neither ketamine nor DREADD-mediated inhibition, or their combination had an effect on social exploration (*p* > 0.05). Consistent with work performed in male (Amat et al., 2016) and female rats (Baratta et al., 2018), IS dramatically reduced social exploration in rats expressing mCherry, relative to mCherry rats that received HC (*p* < 0.01). hM4Di had no effect on IS in rats previously injected with saline. That is, rats targeted with hM4Di that received saline and IS resembled mCherry-expressing rats that received IS and saline (*p* > 0.05). In agreement with the results of Figure 1, prior ketamine, but not saline, administered to rats targeted with PL-DRN mCherry prevented IS-mediated social exploration deficits in rats targeted with PL-DRN mCherry (*p* < 0.01). Strikingly, hM4Di-mediated inhibition of the PL-DRN pathway at the time of IS eliminated the protective effects of prior ketamine. Rats that received this treatment now resembled mCherry rats that received saline before IS or hM4Di rats that received saline before IS (*p* > 0.05).

Although not the focus of the present study, there was significant variability in social exploration for rats that received HC or IS during each phase of estrus (diestrus, proestrus, estrus) for the different treatment groups. However, given the low number of subjects for each stage of estrous in each experimental group, it is not possible to know whether stage of estrous is influencing the behavioral outcome measure (Fig. 6).

## Discussion

The present study set out to determine whether the prophylactic effects of ketamine, as well as the neural mechanisms that mediate its effects, are present in female rats in a manner similar to that which has previously been characterized in male rats (Amat et al., 2016). Thus, we assessed various behavioral and neurochemical endpoints that were examined in previous studies conducted using male rats, while expanding on earlier efforts by assessing specific neural ensembles and circuits implicated in the effects of ketamine. The results of the present experiments provide clear evidence that a single injection of low-dose, but not high-dose, ketamine delivered one week before uncontrollable stress (IS) blocks the behavioral effects of IS on JSE measured 24 h later. These results are in agreement with studies from our laboratory (Amat et al., 2016) and others (Brachman et al., 2016; McGowan et al., 2017) demonstrating prophylactic effects of ketamine in male rats and mice. However, the present study is the first of its kind to demonstrate ketamine prophylaxis in female rats, and suggests that the prophylactic effects of ketamine on later stress outcomes are generalizable to both sexes. This was especially an issue as behavioral control, which has similarities to the effects of ketamine, is not protective in female rats (Baratta et al., 2018), at least under circumstances identical to those used in males (see below).

A significant body of research, primarily derived in male rodents, indicates that behavioral changes produced by IS, including reduced JSE, are mediated by activation of the middle and caudal 5-HT DRN and subsequent release of 5-HT into brain areas such as the BLA (Takase et al., 2004; Amat et al., 2008; Christianson et al., 2010; Dolzani et al., 2016). This intense activation of the DRN is both necessary (Maier et al., 1993, 1994, 1995) and sufficient (Maier et al., 1995) for behavioral changes associated with IS. Thus, we sought to determine whether ketamine administered before IS would mitigate DRN activation typically associated with exposure to IS. Indeed, a single injection of ketamine one week before IS significantly reduced stress-induced activation of the rostral and middle DRN. Although not the focus of the present experiments, it is possible that differences in DRN subregion activation between the female and male studies are due to sex-specific differences in DRN anatomy. While females are reported to have higher basal and stress-induced levels of 5-HT (Domínguez et al., 2003; Mitsushima et al., 2006; Staiti et al., 2011), sex differences in DRN anatomy have received little study. Indeed, one limitation of the present experiments is the use of Fos as the sole marker for DRN 5-HT activation. Indeed, Fos is an immediate early gene that is used to detect recently activated neurons (Sheng et al., 1990) yet unlike *in vivo* microdialysis where extracellular 5-HT can be directly measured, Fos serves as a proxy for DRN 5-HT activation. Even so, the results of the double label IHC experiments strongly suggest that prior ketamine reduces DRN 5-HT activation at the time of stress.

As noted, here we have shown that ketamine blunts both the neurochemical and behavioral consequences of IS when administered one week before the stressor. To date, no other manipulations have demonstrated an ability to produce these effects in female rats. An extensive body of work has revealed that behavioral control over a stressor prevents the neurochemical and behavioral effects of the stressor in male rats (for review, see Maier et al., 2015). Surprisingly, the protective effects of behavioral control are absent in female rats (Baratta et al., in press). The stress-buffering effects of behavioral control observed in male rats have been studied in a paradigm in which rats are provided a controlling response (turning a wheel) over termination of tailshocks (escapable tailshock; ES). A separate group of rats receives an identical series of yoked tailshocks, however, they are unable to control any aspect of the tailshock (IS). Behavioral control blunts DRN activation during the controllable stressor and prevents stress-induced behavioral effects typically measured 24 h later. As previously described, behavioral effects of IS are the result of DRN activation and release of 5-HT into projection regions that are proximal mediators of behaviors associated with the stressor (Grahn et al., 2000; Takase et al., 2004; Amat et al., 2008; Christianson et al., 2010; Dolzani et al., 2016). In male rats, behavioral control activates neurons in the deep Layers (V/VI) of the PL of the mPFC that preferentially synapse on GABAergic interneurons within the DRN (Hajós et al., 1998; Jankowski and Sesack, 2004; Gonçalves et al., 2009). Thus, the net effect of activation of the PL-DRN pathway is reduced DRN 5-HT release and blockade of behavioral effects associated with stressor exposure (Amat et al., 2005). Additionally, experience with ES prevents DRN activation and the behavioral effects of an exposure IS or another stressor such as social defeat one week later (Amat et al., 2006; Amat et al., 2010). Surprisingly, behavioral control fails to activate the PL-DRN pathway in females, and so DRN-mediated behavioral outcomes are not prevented (Baratta et al., 2018). In male rats, the protection afforded by ES against the impact of later uncontrollable stressors is caused by a state change in DRN-projecting PL neurons produced by the initial ES, so that the later experience with IS now activates the PL-DRN pathway (Baratta et al., 2009). Recently, it has also been shown that behavioral control induces plasticity within deep Layer (V/VI) PL neurons that are anatomically situated to prevent DRN activation (Hajós et al., 1998; Varela et al., 2012; Amat et al., 2014). Moreover, blockade of plasticity within the PL with protein synthesis inhibitors, APV, and ERK inhibitors before ES all prevent ES from protecting against the behavioral effects of later IS (Amat et al., 2006; Amat et al., 2014; Christianson et al., 2014). It should be noted that the effects of low-dose ketamine are distinct from those of other NMDA receptor antagonists, such as APV (for review, see Abdallah et al., 2015).[Table T1]

**Table 1. T1:** 

Figure	Data Structure	Type of test	ANOVA *p* values, *t* test *p* values, 95% CI
Fig. 1	Normal distribution	Two-way ANOVA	Main effect of stress: *p* < 0.0001 Main effect of drug: *p* < 0.0001 Interaction: *p* = 0.0249
		Tukey’s multiple comparisons test	
		Homecage:saline vs homecage:ketamine (10 mg/kg)	9−19.55 to 10.49
		Homecage:saline vs homecage:ketamine (40 mg/kg)	−10.27 to 20.6
		Homecage:saline vs stress:saline	7.037 to 37.08
		Homecage:saline vs stress:ketamine (10 mg/kg)	−17.34 to 12.7
		Homecage:saline vs stress:ketamine (40 mg/kg)	6.635 to 37.5
		Homecage:ketamine (10 mg/kg) vs Homecage:ketamine (40 mg/kg)	−5.735 to 25.13
		Homecage:ketamine (10 mg/kg) vs stress:saline	11.57 to 41.61
		Homecage:ketamine (10 mg/kg) vs stress:ketamine (10 mg/kg)	−12.81 to 17.23
		Homecage:ketamine (10 mg/kg) vs stress:ketamine (40 mg/kg)	11.16 to 42.03
		Homecage:ketamine (40 mg/kg) vs stress:saline	1.459 to 32.32
		Homecage:ketamine (40 mg/kg) vs stress:ketamine (10 mg/kg)	−22.92 to 7.942
		Homecage:ketamine (40 mg/kg) vs stress:ketamine (40 mg/kg)	1.067 to 32.73
		Stress:saline vs stress:ketamine (10 mg/kg)	−39.4 to −9.36
		Stress:saline vs stress:ketamine (40 mg/kg)	−15.42 to 15.44
		Stress:ketamine (10 mg/kg) vs stress:ketamine (40 mg/kg)	8.958 to 39.82
Fig. 2	Normal distribution		ANOVA
Total 5-HT Rostral		Two-way ANOVA	Stress: *p* = 0.16 Drug: *p* = 0.47 Interaction: *p* = 0.81
Total 5-HT Middle		Two-way ANOVA	Stress: *p* = 0.59 Drug: *p* = 0.06 Interaction: *p* = 0.60
Total 5-HT Caudal		Two-way ANOVA	Stress: *p* = 0.55 Drug: *p* = 0.23 Interaction: *p* = 0.16
Fig. 2, middle			ANOVA
Total Fos Rostral		Two-way ANOVA	Stress: *p* = 0.0004 Drug: *p* = 0.047 Interaction: *p* = 0.055
		Tukey's multiple comparisons test	
		Saline:HC vs saline:IS Saline:HC vs ketamine:HC Saline:HC vs ketamine:IS Saline:IS vs ketamine:HC Saline:IS vs ketamine:IS Ketamine:HC vs ketamine:IS	−25.9 to −5.303 −10.1 to 10.5 −14.76 to 4.959 5.503 to 26.1 0.8412 to 20.56 −14.96 to 4.759
Total Fos Middle		Two-way ANOVA	Stress: *p* = 0.0004 Drug: *p* = 0.04 Interaction: *p* = 0.044
		Tukey's multiple comparisons test	
		Saline:HC vs saline:IS Saline:HC vs ketamine:HC Saline:HC vs ketamine:IS Saline:IS vs ketamine:HC Saline:IS vs ketamine:IS Ketamine:HC vs ketamine:IS	−17.4 to −3.713 −6.544 to 6.744 −9.418 to 3.251 3.526 to 17.78 0.6301 to 14.31 −9.827 to 3.46
Total Fos Caudal		Two-way ANOVA	Stress: *p* = 0.004 Drug: *p* = 0.62 Interaction: *p* = 0.83
		Tukey's multiple comparisons test	
		Saline:HC vs saline:IS Saline:HC vs ketamine:HC Saline:HC vs ketamine:IS Saline:IS vs ketamine:HC Saline:IS vs ketamine:IS Ketamine:HC vs ketamine:IS	−7.269 to 0.5419 −3.602 to 4.209 −6.341 to 1.069 −0.4295 to 7.763 −3.178 to 4.633 −6.845 to 0.9661
Fig. 2, right	Normal distribution		
5-HT + Fos Rostral		Two-way ANOVA	Stress: *p* < 0.0001 Drug: *p* = 0.046 Interaction: *p* = 0.08
		Tukey's multiple comparisons test	
		Saline:HC vs saline:IS Saline:HC vs ketamine:HC Saline:HC vs ketamine:IS Saline:IS vs ketamine:HC Saline:IS vs ketamine:IS Ketamine:HC vs ketamine:IS	−9.626 to −2.267 −3.43 to 3.929 −5.808 to 1.238 2.516 to 9.876 0.1389 to 7.185 −6.057 to 0.989
5-HT + Fos Middle		Two-way ANOVA	Stress: *p* < 0.0001 Drug: *p* = 0.03 Interaction: *p* = 0.03
		Tukey's multiple comparisons test	
		Saline:HC vs saline:IS Saline:HC vs ketamine:HC Saline:HC vs ketamine:IS Saline:IS vs ketamine:HC Saline:IS vs ketamine:IS Ketamine:HC vs ketamine:IS	−7.904 to −2.494 −2.625 to 2.628 −4.548 to 0.4605 2.382 to 8.019 0.4502 to 5.86 −4.672 to 0.581
5-HT + Fos Caudal		Two-way ANOVA	Stress: *p* < 0.0001 Drug: *p* = 0.03 Interaction: *p* = 0.03
		Tukey's multiple comparisons test	
		Saline:HC vs saline:IS Saline:HC vs ketamine:HC Saline:HC vs ketamine:IS Saline:IS vs ketamine:HC Saline:IS vs ketamine:IS Ketamine:HC vs ketamine:IS	−4.4 to −0.994 −1.515 to 1.892 −3.019 to 0.212 1.099 to 4.672 −0.4097 to 2.996 −3.295 to 0.1108
Fig. 3	Normal distribution		
Total RAM		Two-way ANOVA	Stress: *p* = .99 Drug: *p* = 0.0003 Interaction: *p* = 0.87
		Homecage:saline vs Homecage:ketamine (10 mg/kg) Homecage:saline vs homecage:ketamine (40 mg/kg) Homecage:saline vs stress:saline Homecage:saline vs stress:ketamine (10 mg/kg) Homecage:saline vs stress:ketamine (40 mg/kg) Homecage:ketamine (10 mg/kg) vs homecage:ketamine (40 mg/kg) Homecage:ketamine (10 mg/kg) vs stress:saline Homecage:ketamine (10 mg/kg) vs stress:ketamine (10 mg/kg) Homecage:ketamine (10 mg/kg) vs stress:ketamine (40 mg/kg) Homecage:ketamine (40 mg/kg) vs stress:saline Homecage:ketamine (40 mg/kg) vs stress:ketamine (10 mg/kg) Homecage:ketamine (40 mg/kg) vs stress:ketamine (40 mg/kg) Stress:saline vs stress:ketamine (10 mg/kg) Stress:saline vs stress:ketamine (40 mg/kg) Stress:ketamine (10 mg/kg) vs stress:ketamine (40 mg/kg)	−46.01 to 13.18 −60.26 to −1.071 −27.01 to 32.18 −50.1 to 9.096 −57.58 to −0.5401 −43.85 to 15.35 −10.6 to 48.6 −33.68 to 25.51 −41.16 to 15.88 3.654 to 62.85 −19.43 to 39.76 −26.91 to 30.13 −52.68 to 6.513 −60.16 to −3.123 −37.08 to 19.96
Total Fos		Two-way ANOVA	Stress: *p* < 0.0001 Drug: *p* = 0.1326 Interaction: *p* = 0.1498
		Homecage:saline vs homecage:ketamine (10 mg/kg) Homecage:saline vs homecage:ketamine (40 mg/kg) Homecage:saline vs stress:saline Homecage:saline vs stress:ketamine (10 mg/kg) Homecage:saline vs stress:ketamine (40 mg/kg) Homecage:ketamine (10 mg/kg) vs homecage:ketamine (40 mg/kg) Homecage:ketamine (10 mg/kg) vs stress:saline Homecage:ketamine (10 mg/kg) vs stress:ketamine (10 mg/kg) Homecage:ketamine (10 mg/kg) vs stress:ketamine (40 mg/kg) Homecage:ketamine (40 mg/kg) vs stress:saline Homecage:ketamine (40 mg/kg) vs stress:ketamine (10 mg/kg) Homecage:ketamine (40 mg/kg) vs stress:ketamine (40 mg/kg) Stress:saline vs stress:ketamine (10 mg/kg) Stress:saline vs stress:ketamine (40 mg/kg) Stress:ketamine (10 mg/kg) vs stress:ketamine (40 mg/kg)	−27.09 to 12.92 −34.42 to 5.587 −48.67 to −8.663 −59 to −19 −47.66 to −9.105 −27.34 to 12.67 −41.59 to −1.579 −51.92 to −11.91 −40.57 to −2.021 −34.25 to 5.754 −44.59 to −4.579 −33.24 to 5.312 −30.34 to 9.671 −18.99 to 19.56 −8.657 to 29.9
% RAM + Fos		Two-way ANOVA	Stress: *p* = 0.0064 Drug: *p* < 0.0001 Interaction: *p* = 0.0128
		Homecage:saline vs homecage:ketamine (10 mg/kg) Homecage:saline vs homecage:ketamine (40 mg/kg) Homecage:saline vs stress:saline Homecage:saline vs stress:ketamine (10 mg/kg) Homecage:saline vs stress:ketamine (40 mg/kg) Homecage:ketamine (10 mg/kg) vs homecage:ketamine (40 mg/kg) Homecage:ketamine (10 mg/kg) vs stress:saline Homecage:ketamine (10 mg/kg) vs stress:ketamine (10 mg/kg) Homecage:ketamine (10 mg/kg) vs stress:ketamine (40 mg/kg) Homecage:ketamine (40 mg/kg) vs stress:saline Homecage:ketamine (40 mg/kg) vs stress:ketamine (10 mg/kg) Homecage:ketamine (40 mg/kg) vs stress:ketamine (40 mg/kg)	−21.56 to 5.718 −16.43 to 10.84 −13.64 to 13.64 −40.35 to −13.07 −19.63 to 6.654 −8.516 to 18.76 −5.718 to 21.56 −32.43 to −5.156 −11.71 to 14.57 −10.84 to 16.43 −37.55 to −10.28 −16.83 to 9.452
		Stress:saline vs stress:ketamine (10 mg/kg) Stress:saline vs stress:ketamine (40 mg/kg) Stress:ketamine (10 mg/kg) vs stress:ketamine (40 mg/kg)	−40.35 to −13.07 −19.63 to 6.654 7.084 to 33.37
Fig. 4 Total RB	Normal distribution	Independent samples *t* test T,df	*p* = 0.49 *t* = 0.70; df = 13
Fig. 4 Total Fos	Normal distribution	Independent samples *t* test T,df	*p* = 0.0012 *t* = 4.145; df = 13
Fig. 4*C* % Double Label	Normal distribution	Independent samples *t* test T,df	*p* = 0.0043 *t* = 3.453; df = 13
Fig. 5	Normal distribution	Independent samples *t* test T,df	
Total hM4Di			*p* = 0.83 *t* = 0.21; df = 8
Total Fos			*p* = 0.0025 *t* = 0.4.34; df = 8
hM4Di + Fos			*p* = 0.0037 *t* = 4.05; df = 8
Fig. 6	Normal distribution	Three-way ANOVA	*p* value, power
		Drug Stress Virus Drug*Stress Drug*Virus Stress*Virus Drug*Stress*Virus	0.053, 0.47 <0.0001, 1.0 0.085, 0.39 0.004, 0.851 0.18, 0.252 0.0001, 0.989 0.023, 0.59

Similar to the effects of behavioral control in male rats, a single subanesthetic injection of ketamine enhances synaptic plasticity in PL Layer V (Li et al., 2010) and both prevents (Li et al., 2010; Amat et al., 2016; Brachman et al., 2016; McGowan et al., 2017) and reverses (Li et al., 2011) the effects of stressor exposure. Furthermore, ketamine delivered to male rats induces PL Fos activation (Fuchikami et al., 2015; Amat et al., 2016) and alters PL-DRN neurons so that an experience with IS now activates this pathway (Amat et al., 2016). Given the similarities between the effects of behavioral control and ketamine on later outcomes of IS, we sought to determine whether ketamine alters PL activity in a manner whereby a later experience with IS activates the ensemble of PL neurons initially activated by ketamine. Using RAM, we revealed that low-dose, but not high-dose, ketamine activates ensembles of neurons that are later brought online by IS. This is the strongest evidence to date to suggest that alterations in specific assemblies of PL neurons are involved in the prophylactic effects of ketamine. Others have shown that positive experiences activate neural ensembles in the dentate gyrus and reactivation of these ensembles is sufficient to reverse depression-like behaviors (Ramirez et al., 2015). Future studies should implement similar methodologies to demonstrate necessity of experiential ensembles in the prophylactic effects of prior ketamine.

Given the effect of ketamine on stress-induced DRN activation and PL activity at the time of drug injection and later IS, we sought to determine whether an acute injection of ketamine activates the PL-DRN pathway and whether activation of this pathway at the time of later IS is necessary for the protective effects of the drug. The results of the retrograde tracing study revealed that an acute injection of ketamine activates the PL-DRN pathway. Using a dual virus intersectional genetic strategy, we found that DREADD-mediated inhibition of the PL-DRN pathway at the time of IS prevents the protective effects of ketamine given one week earlier. Others have identified mPFC-hippocampus circuits involved in the acute effects of ketamine given shortly before behavioral testing (Carreno et al., 2016), however this is the first study to reveal a precise neural circuit that mediates the prophylactic effects of ketamine in females. It should be noted that although nonspecific neural and behavioral effects related to CNO metabolism in DREADD experiments have been noted (Gomez et al., 2017), all rats in the present experiment received CNO, and so it is clear that a CNO metabolite is not mediating the observed effects on behavior.

Taken together, the present experiments indicate that ketamine exerts long-lasting prophylactic effects against the deleterious outcomes of stress in female rats. The effects of ketamine persist much longer than the plasma half-life of ∼2 h in rats (Williams et al., 2004), and so the observed effects cannot be attributed to acute drug effects at the time of behavioral testing. The pattern of the data suggests that the effects of ketamine mirror the long-lasting prophylaxis that occurs in male rats provided with behavioral control. This data suggests that while behavioral control fails to protect female rats, the necessary resilience circuitry does exist and is engaged by ketamine. Therefore, ketamine may prove effective as a therapeutic strategy for female clinical populations likely to experience high levels of stress or trauma.

## Synthesis

Reviewing Editor: Jorge J. Palop, Gladstone Institutes and UCSF Decisions are customarily a result of the Reviewing Editor and the peer reviewers coming together and discussing their recommendations until a consensus is reached. When revisions are invited, a fact-based synthesis statement explaining their decision and outlining what is needed to prepare a revision will be listed below.

These are a few suggestions:

- Justify in the abstract the clinical relevance of performing these experiments in females.

- Add in the figure legends the statistical tests for the indicated asterisks in the graphs.

- Be consistent with labels in the graphs and text. For example, Fos-ir cells, Fos+ cells, or Fos are indistinctly used in the figures. Please also be consistent with the y-axis labels, both ‘Fos+’ or ‘Fos-ir cells’ are used. Add ‘cells’ if ‘cells’ is the unit. For the behavior, Figure 1 uses ‘Homecage’ and ‘Stress’ but Figure 6 uses ‘HC’ and ‘IS’.

- Figure 1, the results and figure legend describe high (40 mg/kg) and low (10 mg/kg) doses of ketamine, but Figure 1 only shows one ketamine-treated group. Please fix this.

- The stats in Figure 1 do not match. There are three asterisks in the graph and one asterisk in the legend. Legend says ‘compared to HC’ but graph shows the ketamine comparisons. Please revise this figure so results, legend, and graphs match.

- Figure 2, indicate the dose of ketamine in the results. Is this the high or low dose?

- Figure 3, add the panel letters (A, B, C, D, and E) to indicate the panels.

- Figure 3C, eYFP or GFP? Please match text and legend. Scale bar is mentioned but not indicated.

- Figure 3D, please describe the panel with yellow and white arrows before the quantification.

- Figure 4, add the panel letters (A, B, C, D, and E) to indicate the panels. Please match the stats, **** are missing in the legend.

- Figure 5, please check if the scale bar is 50 um as indicated in the legend.
